# You Need to Know: evaluation of a uterine cancer awareness campaign for Black and Asian ethnic minority women in North East London

**DOI:** 10.1136/bmjph-2024-002475

**Published:** 2025-09-18

**Authors:** Anisha Chitrakar, Natalie Darko, Nessa Millet, Sherrice Weekes, Harjeet Dhanota, Alexandra Lawrence, Caroline Cook, Esther L Moss

**Affiliations:** 1 College of Life Sciences, University of Leicester, Leicester, England, UK; 2 Department of Gynaecological Oncology, University Hospitals of Leicester NHS Trust, Leicester, England, UK; 3 North East London Cancer Alliance, NHS North East London, London, England, UK; 4 Department of Gynaecological Oncology, The Royal London Hospital, London, England, UK

**Keywords:** Community Health, education, Female, Communication

## Abstract

**Introduction:**

The ‘You Need to Know’ (YNTK) uterine cancer (UC) awareness campaign was designed and conducted in North East London (NEL) in response to high UC mortality rates reported among women from Black and Asian minority groups. YNTK aimed to raise awareness of red-flag symptoms, address misconceptions and encourage earlier presentation to healthcare services with a long-term aim of reducing the number of patients diagnosed with advanced stage UC. Information materials were designed in consultation with members of Black and Asian ethnic minority groups from NEL, and the YNTK campaign was delivered between January 2023 and April 2024, in collaboration with The Eve Appeal, a gynaecological cancer charity.

**Methods:**

A process evaluation was conducted using data collected through (1) A questionnaire of pre-campaign UC awareness in women from the target population, (2) Outreach talk attendance and feedback questionnaires and (3) Interviews with staff members who played a role in organising or delivering the outreach talks.

**Results:**

Application of the reach, effectiveness, adoption, implementation and maintenance (RE-AIM) framework enabled a comprehensive analysis of the impact of YNTK, and enabled the identification of areas for campaign refinement. In particular, enhancing language interpreter support and overcoming barriers to in-person events were highlighted. It was acknowledged that the campaign did require resources in the form of staff and venues, and maintenance could be supported through the use of multi-lingual information resources shared by community members.

**Conclusions:**

The YNTK campaign has provided valuable insights into maximising the effectiveness of a targeted cancer awareness intervention at the community level. Despite logistical challenges, YNTK appears to have been effective in achieving its primary objective of increasing UC awareness, as evidenced by the positive feedback from event attendees, although the clinical impact on UC diagnosis will need to be evaluated over the coming months and years.

**WHAT IS ALREADY KNOWN ON THIS TOPIC**

- National cancer awareness campaigns are reported to have limited reach within underserved and minority populations, whereas community-based interventions can be effective in promoting health awareness.

**WHAT THIS STUDY ADDS**

- The ‘You Need to Know’ uterine cancer awareness campaign was designed to address inequities in UC outcomes by using a community-centred approach that actively engaged community members, healthcare providers and a gynaecological cancer support charity in crafting culturally relevant health messaging to increase relevance and appropriateness.

**HOW THIS STUDY MIGHT AFFECT RESEARCH, PRACTICE OR POLICY**

- Targeted and culturally-tailored campaigns have the potential to raise cancer awareness amongst ethnic minority women in underserved communities. However, sustained long-term funding support and engagement with communities will be needed to extend the reach to larger populations.

## Introduction

 Uterine cancer (UC) is now the fourth most common female malignancy in the UK,^1^ and its incidence is continuing to rise. Although UC is viewed as a malignancy with a good prognosis, recent data from the UK demonstrates stark variation in survival,^2^ disproportionately affecting women from ethnic minority and socioeconomically deprived subgroups.^3^ In particular, women from Black ethnic minority backgrounds have been shown to experience the highest mortality rates, which has been attributed to higher rates of advanced-stage disease at diagnosis.^4^ Reasons for these findings include barriers to accessing healthcare, as well as low levels of awareness of red-flag symptoms, normalisation or minimisation of symptoms and social stigmas surrounding gynaecological conditions.^5^,^7^

Community-based interventions can be effective in promoting health and screening among different populations; for example, a community-based intervention using educational workshops, community health workers and tailored materials to promote cervical cancer screening among Vietnamese-American women resulted in increased screening coverage.^8^ Key aspects of such programmes include cultural awareness and sensitivity, educators who can overcome language barriers and partnerships with the target populations.^9 10^ In the UK, targeted cancer awareness campaigns aimed at specific populations have been shown to have impact, as exemplified by the prostate cancer campaigns aimed at Black men, for example, ‘Play Domino, Talk Prostate’.^11^ These programmes appear to having an impact with Black men resident in the UK reported to be less likely to present with advanced prostate cancer compared with men from other ethnic groups.^4^

### Campaign development

**Figure 1:**
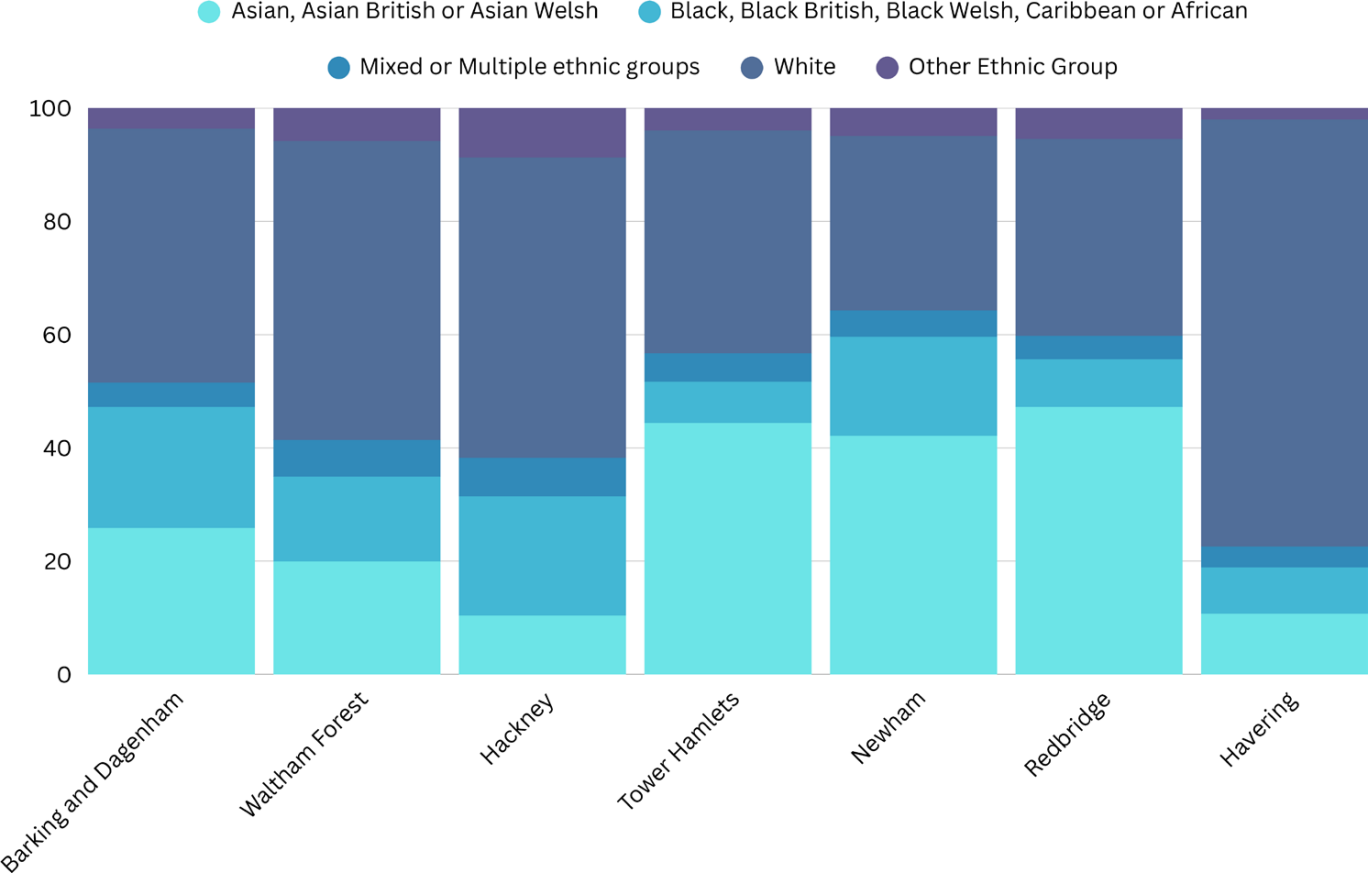
Ethnically diverse population in North East London by borough. Percentage of residents, data from the Office for National Statistics.^10^

The need for a UC awareness campaign was a result of growing concern among clinical teams working in East London due to the high proportion of patients of Black African ethnicity presenting with late-stage UC, and consequently having significantly worse survival compared with patients from other ethnic groups. A particular concern was the lack of awareness among women in the local population that postmenopausal bleeding was a sign of UC, and should prompt a medical review. As a result, a collaboration between The Eve Appeal and North East London Cancer Alliance (NELCA), whose geographical area covers Waltham Forest, Redbridge, Barking and Dagenham, Havering, Newham, Tower Hamlets, Hackney and City of London, highly ethnically diverse locations, was developed to undertake a UC awareness campaign focusing on women from Black and Asian populations (figure 1). The objective of the campaign was to increase awareness of UC, with the long-term aim of decreasing the proportion of late-stage diagnoses and, as a result, improving mortality among women from Black and Asian populations, who are historically inadequately served by conventional health promotion approaches.

A health communications company was recruited to work with the team to develop the campaign alongside NELCA, with a particular focus on cultural sensitivities and crafted to be relatable to the diverse community of NEL. In the development phase of the campaign, women from different communities were recruited to work collaboratively with the team to guide and be consulted on the design of the campaign materials. This was achieved through focus groups, ensuring the content and language used were appropriate and resonated with the largest communities’ experiences and perspectives. The title for the campaign ‘You Need to Know’ (YNTK) was chosen through focus group discussions and the key message was determined to be ‘Bleeding after the menopause is not normal’. The word ‘womb’, rather than uterus, was preferred since this was deemed to be a more widely known term among lay audiences.

### You Need to Know campaign

The YNTK campaign consisted of several components, including community outreach events, dissemination of printed information leaflets (available in five languages: English/Bengali/Urdu/Somali/Gujarati), and social media awareness. The community outreach events were arranged through networking contacts in community groups by NELCA, and the talks were delivered by The Eve Appeal advocacy consultant. The event format consisted of an information presentation, approximately 45–60 min, followed by a 30 min group discussion and a question/answer session.

The YNTK campaign was launched on 31 January 2023, with 12 community outreach events taking place between February 2023 and April 2024. Ten of the events were in-person, taking place in all the NEL boroughs except Havering, and were mainly held on weekdays within office hours, whereas the two virtual events were held using the Zoom platform in the evening. In addition, posters were displayed in the local community centres and event venues, and information on YNTK was disseminated through social media and the NELCA YouTube channel (https://www.youtube.com/watch?v=WQJBfTHZzb4).

### Campaign evaluation

There is a growing need for multicomponent public health campaigns to be evaluated to understand how such campaigns do, or do not, have an impact in order to refine future implementation.^12^ In particular, evaluations can uncover the processes underpinning the experiences of those delivering and receiving the campaign to ascertain its acceptability. The aim of this study was to conduct a process evaluation of YNTK to understand how the campaign was delivered and how it might have had an impact, with a particular focus on exploring future avenues for refinement.

## Methods

Evaluation of the YNTK campaign was conducted through a process evaluation using the reach, effectiveness, adoption, implementation and maintenance (RE-AIM) framework^13^ and following the Medical Research Council guidance on evaluating complex interventions.^12^ The study was sponsored by the University of Leicester and ethical approval was granted by the Health Research Authority (23/IEC08/0003). Participants who completed the online questionnaire gave consent through agreement with consent statements before completing the questionnaire. Written informed consent was offered and obtained, where possible, for the completion of the anonymous outreach event questionnaires. All interview participants gave written informed consent.

### Data collection and analysis

#### Pre-campaign awareness

For the pre-campaign questionnaire, a UC awareness questionnaire covering key diagnosis and symptom information was created through an iterative process with the study team and healthcare professionals involved in the diagnosis and care of patients with UC (online supplemental data 1). A link for the questionnaire, using the online survey platform *JISC*, was disseminated through social media channels (Facebook, Twitter and Instagram) 2 weeks prior to the launch of the campaign. Descriptive statistics for feedback questionnaire outcomes and UC awareness scores are presented.

#### Campaign implementation

To understand how the campaign was implemented and whether there were any deviations from planned implementation, data was recorded on: the number of outreach events that took place; and the number of attendees at each event, along with associated demographic characteristics of attendees. Interviews with all staff members who played a role in organising or delivering the outreach talks were conducted to explore their experience of the campaign delivery and logistical challenges that impacted implementation. To understand how the campaign did, or did not, have impact, attendees at the outreach events were invited to complete a feedback questionnaire to capture their views on the talk content and potential impacts on health-seeking behaviour (online supplemental data 2). No sample size calculations were performed; however, all key personnel involved in the design, planning and implementation of the study were interviewed.

The semi-structured interviews were conducted after the completion of the campaign (April – July 2024) by a researcher (AC) trained in qualitative research methods and followed an interview guide exploring the training, resources, adaptations and challenges that occurred prior to and during the campaign (online supplemental data 3). Interviews were conducted virtually using Microsoft Teams, and field notes were taken. Interviews were recorded and transcribed using transcription software. Interviews and free-text quotes from the questionnaires were analysed using a reflexive thematic analysis.^14^ Researchers AC and ND independently coded interview transcripts and questionnaire responses; iterative team discussions refined initial codes into themes, and the RE-AIM framework was used to structure the identified themes.

## Results

### Pre-campaign awareness

Although the response to the online questionnaire was low (23 participants), the majority belonged to the campaign’s target audience (online supplemental data 4). Only 43.5% of respondents were aware of UC red-flag symptoms, and many reported a lack of urgency in seeking medical attention should they experience symptoms, 17.4% within 1–2 weeks, and 13% within 1–6 months. Over half of the respondents (56.5%) held the misconception that a cervical smear test was able to screen for UC. Potential barriers to accessing healthcare for red-flag symptoms were also reported, for example, the sex of the healthcare professional was identified as a barrier to accessing care, with one respondent stating: *‘I would only attend if I could be guaranteed to see a female doctor’*.

**Figure 2:**
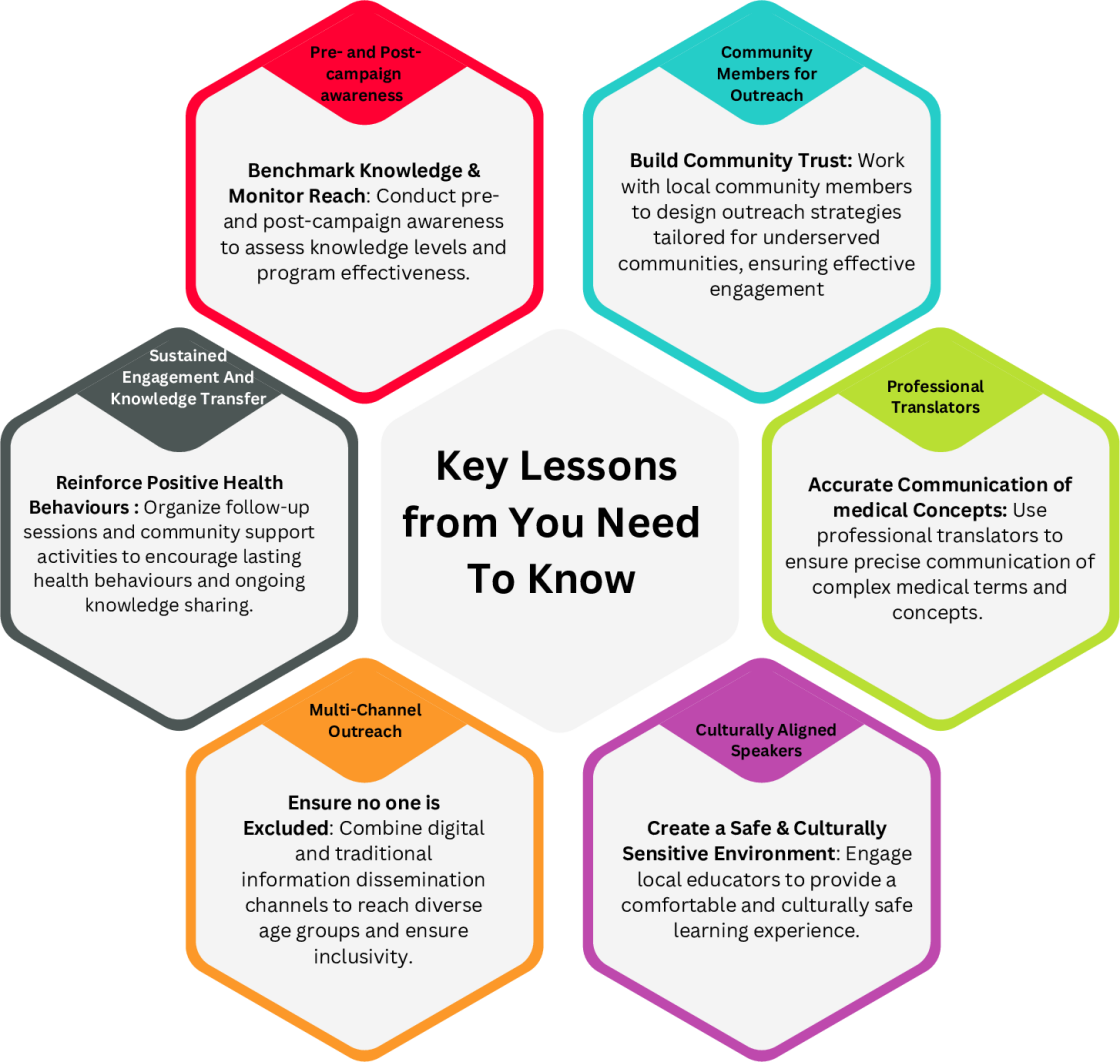
Key lessons from the You Need to Know campaign.

The RE-AIM framework was used to identify key lessons from the YNTK campaign (table 1, figure 2 and online supplemental data 5).

**Table 1:** ‘You Need to Know’ campaign: summary of the process evaluation using the reach, effectiveness, adoption, implementation and maintenance framework

Dimensions	
**Reach**	**Demographics:** 88% of attendees were women above 45 years; 85.7% were from Black or Asian backgrounds **Engagement challenges:** engaging the community was challenging: …trying to get into these community places… took a lot of emails…(they) don’t know what you’re asking them for’
**Effectiveness**	**Understanding:** 92.8% found information easy to understand; 97.6% felt symptoms information was adequate **Behavioural impact:** 92.8% were encouraged to consult healthcare professionals; 94% intended to share knowledge: ‘*I will share with known and family*’ **Cultural insights:** the campaign addressed misconceptions, such as the difference between cervical and womb cancer, and the role of screening: ‘*…women were under the impression that the smear test tested for womb cancer*’
**Adoption**	**Community engagement:** despite challenges, media engagement efforts included a TikTok reel with 12.9K views, and anatomy props to enhance understanding. Language barriers persisted: ‘*…language is one of the biggest barriers*’ **Speaker delivery:** feedback was overwhelmingly positive: *‘…the talks were very gifted with…a great way of communicating…very positive*’
**Implementation**	**Session quality:** 89.7% found the location convenient, and 91.2% felt the session length was appropriate. Participants remarked on the session’s quality: ‘*…all positive. I always left feeling like the session had gone well*’ **Knowledge sharing:** participants valued shared experiences and cultural relatability: ‘*A lot of women said what a useful time they had…they would share the learning with their friends and family*’
**Maintenance**	**Future expansion:** plans to include other gynaecological cancers, with participants indicating interest in continued sessions: ‘…*we’re thinking of how we can expand it to talk about ovarian cancer*’

### Reach

In total, 250 individuals attended an event. The median number of attendees per event was 25, with a range between 1 and 40 individuals (table 2), with no difference in attendance between the in-person and virtual events, p=0.85. Event evaluation questionnaires were completed by 37.9% of the attendees at the in-person and 13.6% of the virtual events. The majority of those who completed a feedback questionnaire were from the campaign’s target audience, with 88% identifying as female and over 45 years, and 85.7% were of Black or Asian ethnicity. In total, nine people participated in an interview. Data from the interviews suggested that the event location influenced the audience, for example, an event in a Gurdwara attracted women of Asian ethnicity, whereas the audience at an event in a church was predominantly women of Caribbean heritage. Events at non-religious locations, for example, a menopausal café and a GP surgery, also attracted a large ethnic minority audience. It was noted that some men had attended because the locations of some of the talks were accessible to all:

‘*…*sometimes the groups were (mixed), sometimes they were women, specific groups, like at the temple we went to together. And then sometimes they were more general community groups where it was open to everybody’ (YNTK team)

**Table 2:** Demographic characteristics of You Need to Know event attendees who complete an evaluation questionnaire

	Face-to-face events	Virtual events
Sex		
Female	87.2%	100%
Male	5.1%	0%
Not disclosed	7.1%	0%
Sexual orientation		
Heterosexual	64.1%	100%
Gay and Lesbian	0%	0%
Bisexual	0%	0%
Don't know	0%	0%
Prefer not to say	36%	0%
Age		
>55 years	36%	33.3%
45–54 years	19.2%	33.3%
40–44 years	5.1%	16.7%
<40 years	5.1%	0%
Not disclosed	34.6%	16.7%
Ethnicity		
Black, Black British, Black Welsh, Caribbean or African	39.7%	100%
Asian, Asian British or Asian Welsh	45%	0%
Mixed or multiple ethnic group	2.6%	0%
White	6.4%	0%
Prefer not to say	6.4%	0%
Other ethnic group	0%	0%
Religion		
Christian	50%	83.3%
Muslim	16.6%	0%
Hindu	9%	0%
Sikh	12.8%	0%
Jewish	1.3%	0%
Not specified	10.3%	16.7%

The role of a community engagement lead was seen as vital to using connections and trust with different organisations within the community. It was acknowledged that this approach took more time than anticipated; however, it was agreed that having a collaborative approach and engaging with communities was a significant factor contributing to the success of YNTK.

### Effectiveness

The evaluation questionnaires identified that the campaign appeared to have the desired impact on attendees, with 92.8% of the respondents reporting that they found the factual information easy to understand and appropriate, and attendance had encouraged them to talk to a healthcare professional (92.8%) and their friends and family (94%) about UC symptoms. Some of the comments revealed attendees’ previous experiences and knowledge, for example, *‘had bleeding last year, was seen and checked and was told nothing serious since had womb cancer, so aware of it’* (Event attendee). Whereas for other participants, the talk resulted in knowledge acquisition: *‘It makes me more aware of what is happening in my body’* (Event attendee).

Although 97.6% of the respondents reported that the information on the symptoms was adequate, some comments did raise concerns regarding comprehension of the health messaging and knowledge of reproductive anatomy, since one attendee said, ‘*It is a fascinating topic and very much educational about women’s cancer awareness. I enjoyed listening to discussions about women’s vagina cancer’* (Event attendee). The need to iteratively develop the information content of the talks to include an explanation of gynaecological anatomy and menopause was identified by the Advocacy Consultant in the initial events as a result of audience questions: *‘Some ladies commented on how the props help them learn about Gynaecological anatomy. There was one female who wasn’t aware of a perimenopausal stage before’* (YNTK team). In addition, the need to address misconceptions, in particular, the view held by many attendees that cervical smear tests could be used to detect all gynaecological cancers, led to the provision of information to counter this specific issue at every event.

### Adoption

The essential role of advocates within the communities to support the campaign and the use of social media to raise awareness of events was acknowledged. Despite consulting with community representatives during the development of the campaign materials, members of the organising team felt that it had been a challenge to identify outreach locations for the talks and to raise interest within the target audience:

‘The most challenging was actually trying to get into these community places. So, it took a lot of emails, a lot of once you did get through to them, and you have to have a core to even pitch the talk because they don’t know what you’re asking them for’ (YNTK team)

Additional logistical barriers, including budget limitations and a lack of familiarity with working with a health communications company, were reported, although the greatest concern was trying to address the requirements and expectations of the diverse ethnic and cultural groups.

Language, in particular, was identified as a barrier to both the potential reach and adoption of the campaign. The multilingual leaflets were identified as an important resource, but it was acknowledged by staff that translation services would also benefit the accessibility of the talk delivery.

‘I’ve got the flyers and the assets in different languages, but where English is not their language, I felt maybe a translator would have been a good idea …because you want to have a really good impact… a full impact of the discussion. I think that language is one of the biggest barriers’ (YNTK team)

It was felt that the ethnic background of the speaker could also potentially influence the audience, and that involving women from Black and Asian groups and communities to help deliver the talks could add to the impact of future events. *‘I think sometimes people have said that they would really value somebody from their own community delivering those talks’* (YNTK team).

### Implementation

The majority of staff involved with YNTK felt that the campaign had achieved the campaign’s objective of raising UC awareness within the target audience. The majority (89.7%) of the respondents reported that they found the location of the talk was convenient, with only 10.3% thinking that the location was not appropriate. Informal feedback to the staff from attendees was positive, and they were repeatedly told how informative and enjoyable the events were and how grateful the community was:

‘Everyone was grateful for the presentation and their increased knowledge was reflected in the evaluation feedback’ (YNTK team)

This was supported by the respondents, who also highlighted a strong sense of community and involvement, even when facing logistical issues, such as clashes with other planned activities within the community.

### Maintenance

All the staff felt that the campaign could be maintained and that continuation was necessary in order to extend its reach at the community level. *‘I think being able to continue the programme for longer and being able to offer more talks would be really good as well, and it may be that we have run a few more’* (YNTK team).

The potential for transferring the learning and experiences from YNTK to raise awareness of other cancers and campaigns was also discussed by staff: *‘We’re obviously thinking about phase two now and thinking about what else, what other cancers female get, that we can include, we’re in discussion with that already’* (YNTK team).

Continuing to engage and educate the audience was seen as an important next step, including additional information to address audience questions on the aetiological reasons for UC disparities and widening the scope to include other medical conditions. Specifically, *‘they wanted breast cancer awareness and bowel cancer as well’* (YNTK team).

It was acknowledged that the campaign did require resources in the form of staff and venues, and maintenance could be supported through the use of multi-lingual information resources shared and developed by community members.

### Real-world impact

Rapid cancer registration data for NEL has shown a change in the percentage of UCs diagnosed at an early stage from 70% in February 2023, at the start of the YNTK campaign, to 77% in July 2024. Further evaluation will be needed to explore whether YNTK played a role in this change, however, the trend is encouraging.

## Discussion

The YNTK campaign was designed to address inequities in UC outcomes by using a community-centred approach that actively engaged community members, healthcare providers and a gynaecological cancer support charity in crafting culturally relevant health messaging to increase relevance and appropriateness.^15^ By targeting health knowledge deficits and addressing psychosocial aspects related to UC symptoms and aetiology, YNTK exemplifies a culturally tailored health promotion model aimed at reducing health disparities.^16^ This campaign was novel in its approach since it primarily sought to engage with communities in person and not through social media or press outreach, as has been the approach in other campaigns, for example, #RedDabRedFlag. This approach is also supported by evidence from national cancer awareness campaigns, such as ‘Be Clear on Cancer’, which have struggled to reach underserved and minority populations, highlighting the importance of culturally-tailored methods to improve engagement and access.^17^

Although YNTK was developed in response to a clinical imperative rather than a formal academic theory, it encompasses principles within the health belief model (HBM), which has been demonstrated to be effective in cancer screening and awareness programmes. More specifically, the campaign addresses perceived susceptibility, severity, barriers and benefits, besides giving cues to action through its intent to empower the participant in health-seeking behaviours.^18^ Additionally, the HBM can be applied in a culturally tailored or culturally sensitive way by researchers and practitioners who can adapt its constructs (eg, perceived susceptibility, severity, barriers, benefits) to address the unique needs, values and cultural contexts of specific populations.^19^

The pre-campaign evaluation of YNTK revealed low levels of awareness of UC red flag symptoms, with widespread misconceptions. The response rate to the online questionnaire was low, despite dissemination through the community, online groups and social media. Potential reasons for this include that people may be reluctant to respond to an unsolicited online questionnaire, and in-person data collection, for example, before an outreach event, may result in greater engagement and a higher response rate. Also, English language proficiency and digital exclusion are other known barriers that could have contributed to the response rate. Similarly, the evaluation of the campaign highlighted challenges in gaining feedback at the outreach events. A participant information sheet and written consent form were offered prior to completion of the anonymous evaluation and demographic questionnaires; however, the completion of the forms was inconsistent and made more challenging given the number of attendees, literacy levels and the duration of the event. Completion of the feedback form and demographic form was taken as implied consent. We have demonstrated that a written consent process in this type of research study and population serves little value, and that future evaluations should consider different forms or methodologies in order to obtain consent.

A UC awareness measure was recently validated in a predominantly White, highly educated population^20^ and highlights the lack of assessment tools designed for underserved populations that may not have English as their primary language. It is therefore important to develop and validate a culturally sensitive, multilingual awareness measure for the target audience of YNTK, since health literacy and language barriers can greatly affect understanding and engagement.^15^ The study by Singleton and Krause, underlined how health literacy and linguistic barriers burden UC awareness and further pressed for culturally adapted awareness-raising materials to enhance awareness and stimulate health-seeking behaviour across diverse populations.^21^

The primary objective of YNTK was to increase knowledge of red-flag symptoms at the community level to encourage earlier UC presentation and diagnosis. Community-based campaigns have been shown to successfully use culturally acceptable messaging to encourage peer dissuasion as a strategy for promoting positive health knowledge and attitude change.^22^ The collaborative design of YNTK reflects the principle of reciprocal determinism, whereby individuals and their social environments influence each other. Participation by those targeted in the campaign can engender a sense of ownership for the campaign messages and nurture positive health behaviour.^23^ Evidence indicates that open health discussions within a culturally supportive context serve to reduce stigma and increase help-seeking behaviour among underserved groups.^24^ This approach could be expanded at scale through the training of community ambassadors,^25 26^ which have been shown to be successful within other culturally tailored health programmes for sustaining positive health behaviours.^26^ The creation of culturally tailored information resources that could be used to ensure the factual accuracy of the health messaging, for example, the ‘Seeing red…?’ information video,^27^ with different language versions overcoming the requirement for interpreters.

The YNTK campaign achieved its aim of engaging women from a target audience, although the necessity for physical attendance at events was cited as potentially creating barriers to accessibility for some individuals or groups. Younger audiences are reported to have higher engagement through digital events; however, older audiences tend to prefer an in-person approach through trusted community ambassadors.^28^ YNTK therefore may benefit from a greater hybrid outreach model using digital platforms to reach younger audiences, while ambassador-led, in-person sessions could be aimed at older age community members.

The importance of language and the need to overcome health literacy challenges were key messages that emerged from the YNTK evaluation, reflecting the linguistic and educational diversity of NEL. Evidence to date suggests that interpreters and the development of multilingual materials facilitate health knowledge in linguistically diverse settings.^15^ However, even medical terminology presents a considerable challenge when translating across groups, given that lay groups may not be familiar with anatomical terms and clinical language. YNTK used anatomical models to enhance understanding, and this is supported by previous studies that have used visual aids to improve comprehension and reduce miscommunication of information.^29^ Visual displays have also been used in conjunction with culturally appropriate messaging to improve comprehension, especially in communities with limited health literacy levels.^30^

Application of the RE-AIM framework has enabled a comprehensive analysis of the reach, effectiveness, adoption, implementation and maintenance of YNTK, and has helped to identify areas for improvement.^31^ For example, additional information on reach and adoption could be improved by using social media analytics to monitor engagement and by tracking changes in the number of primary care appointments for UC symptoms for women in the targeted populations.^32^ Effectiveness could be further assessed through a more extensive pre-campaign evaluation and adding a post-campaign survey, capturing changes in knowledge and symptom awareness. The collection of qualitative feedback from community leaders and healthcare providers could also enrich the evaluation. Incorporating specific behavioural change metrics, such as the number of ambassador-led community interactions, could provide additional insight into sustained engagement.^33^ Another approach would be to undertake Ripple Effects Mapping,^34^ which visualises programme impact through focus group data. This was initially considered for the YNTK evaluation, but could not be undertaken due to the limited number of attendees in the different locations.^34^ Nonetheless, gathering alternative qualitative data, such as testimonials or healthcare provider feedback, could provide valuable insights into YNTK’s impact at the community level.^35^

Future evaluations could be further developed and scaled up by integrating the practical, robust implementation and sustainability model (PRISM) framework, which emphasises the need to conceptualise, assess and address contextual domains with a strong focus on health equity.^36^ By increasing contextual responsiveness, the PRISM framework allows for better alignment with community-specific needs before, during and after implementation efforts.^37^ For instance, key contextual domains such as community infrastructure, healthcare access and cultural norms can be systematically evaluated to ensure that the intervention aligns with local realities. Additionally, the PRISM framework promotes iterative adaptations, enabling ongoing adjustments to maximise responsiveness to the needs of marginalised groups and reduce inequities in cancer awareness and care. This could involve engaging with community leaders and stakeholders at all stages to co-design equitable solutions, ensuring that barriers such as language access and cultural appropriateness are consistently addressed.

Moreover, incorporating the PRISM framework could enhance scalability by embedding a structured approach to assess sustainability within diverse settings. Jolles *et al*,^37^ highlighted the importance of integrating real-time contextual feedback to enhance alignment with local contexts and support health equity. For example, the inclusion of context-specific equity indicators, such as healthcare access metrics or community engagement levels, would ensure that scaling efforts do not inadvertently widen disparities. Evaluations could also incorporate equity-focused feedback loops, using real-time data to adapt strategies dynamically, thereby maintaining alignment with evolving community needs.^37 38^

## Conclusions

The YNTK campaign has provided valuable insights into maximising the effectiveness of a cancer awareness intervention at the community level. Its tailored approach, focusing on women from Black and Asian ethnic minority groups and communities, was a key factor in achieving its objectives. The use of multilingual leaflets helped to ensure that information was accessible across languages and could be shared within communities with low levels of English language proficiency. The information delivered aimed to raise awareness and address misconceptions with the aim of improving individuals’ knowledge and engagement with healthcare services. Despite logistical challenges, YNTK appears to have been effective in achieving its aims, as evidenced by the positive feedback from event attendees. However, further work is needed to extend the reach of the campaign to larger populations and to more effectively engage community members to propagate the health messaging. A long-term funding base will also be essential and should include the creation of additional multi-lingual resources. The true impact of YNTK will only become evident over time when the longitudinal impact on UC mortality can be measured, and the recently established Endometrial Cancer Audit Pilot will be a potential method of monitoring the impact across England.^39^ However, the lessons learnt from the campaign are already being used by the NELCA by extending the campaign to include ovarian cancer (https://www.nelcanceralliance.nhs.uk/youneedtoknow-ovarian).

## Supplementary material

10.1136/bmjph-2024-002475 online supplemental file 1

## Data Availability

No data are available.
