# Supplementary material for: You Need to Know: evaluation of a uterine cancer awareness campaign for Black and Asian ethnic minority women in North East London
Source: BMJ Public Health. 2025 Sep 18;3(2):e002475. doi: 10.1136/bmjph-2024-002475 (PMC12458616; doi:10.1136/bmjph-2024-002475)
Supplement: online supplemental file 1 [file bmjph-3-2-s001.docx]

**Supplementary Data 1: The Uterine Cancer Awareness Questionnaire**

1. Do you know of any warning signs of womb cancer? Please name as many as you can in the space below:
2. The following may or may not be warning signs of womb cancer. We are interested to know whether you think each of the following is a sign of womb cancer:
3. Vaginal bleeding between periods
4. Persistent lower back pain
5. Persistent abdominal pain
6. Persistent vaginal discharge that smells unpleasant
7. Discomfort or pain during sex
8. Menstrual periods that are heavier or longer than usual
9. Bowel issues
10. Vaginal bleeding after the menopause
11. Blood in the stool or urine
12. A lump or swelling in your tummy or pelvis
13. Losing weight without trying
14. If you had a symptom that you thought might be a sign of womb cancer, how soon after it started would you contact your doctor to make an appointment to discuss it?
15. In the next year, who do you think is most likely to develop womb cancer in the UK?
16. What things (risk factors) do you think affect a person’s chance of developing womb cancer?
17. The following things (risk factors) may or may not increase a person’s chance of developing womb cancer. Some of these are correct and some of these are myths. How much do you agree that each of these can increase a person’s chance of developing womb cancer?
18. Smoking cigarettes
19. Never using the contraceptive pill
20. Not having children
21. Being overweight
22. Having Polycystic ovary syndrome (PCOS)
23. Going through the menopause over the age of 55
24. Having diabetes
25. Having many sexual partners
26. Having breast cancer treatment (Such as Tamoxifen)
27. Rarely exercising
28. Having relatives with bowel cancer or womb cancer
29. Hormone Replacement Therapy
30. How confident are you that you would recognise a womb cancer symptom?
31. Do you think that a cervical smear test screens for womb cancer?

**Supplementary Data 2: Outreach talks evaluation questionnaire**

**Please place a tick in the box that is most true for**

1. **Please rate how much you agree with the following statements?**

|  |  | **Strongly agree** | **Agree** | **Neither agree or disagree** | **Disagree** | **Strongly disagree** |
| --- | --- | --- | --- | --- | --- | --- |
|  | The location of the talk was convenient | **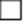** | **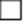** | **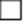** | **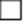** | **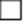** |
|  | The session was enjoyable | **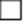** | **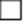** | **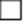** | **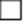** | **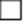** |
|  | The level of the session was appropriate (i.e. the information was easily understandable) | **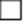** | **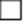** | **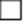** | **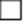** | **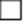** |
|  | The length of the session was appropriate | **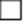** | **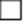** | **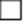** | **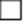** | **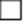** |

1. **Do you agree that the talk provided you with adequate information on the symptoms of womb cancer?**

|  | Strongly agree | **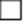** |
| --- | --- | --- |
|  | Agree | **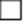** |
|  | Neither Agree or Disagree | **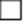** |
|  | Disagree | **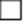** |
|  | Strongly Disagree | **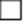** |

1. **Do you agree that the session has encouraged you to talk to a health professional if you notice any symptoms relating to womb cancer?**

|  | Strongly Agree | **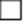** |
| --- | --- | --- |
|  | Agree | **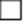** |
|  | Neither Agree or Disagree | **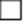** |
|  | Disagree | **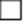** |
|  | Strongly Disagree | **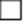** |

1. **Do you agree that the session made you want to speak to friends and family about the symptoms of womb cancer?**

|  | Strongly Agree | **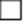** |
| --- | --- | --- |
|  | Agree | **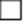** |
|  | Neither Agree or Disagree | **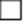** |
|  | Disagree | **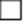** |
|  | Strongly Disagree | **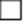** |

**Supplementary data 3: Semi-Structured interview guide**

Introduction and welcome

1. What was your experience of delivering the outreach talks?

- (PROMPT depending on answer)

1. Were there any aspects that you particularly enjoyed about delivering the talks? PROMPT= Why?
2. Did you find that the talks were challenging to deliver in anyway? PROMPT= Why?
3. Was there any comments or discussion with audience during your talk?
4. Were all of the talks the same in terms of content and structure, or did they differ in any way?

- How did they differ?
- Why was this necessary

1. How did you feel the attendees perceived the talks? Why do you think this?
2. What feedback did you receive on the talks?
3. Are there any ways that the talks could be improved to be delivered in this community?

**Supplementary Data 4. Demographic characteristics and responses of YNTK pre-campaign uterine cancer awareness and knowledge (n=23)**

|  | Percentage |
| --- | --- |
| Sex  Female  Prefer not to answer | 91.3%  8.7% |
| Sexual orientation | Heterosexual or straight: 91.2%  Gay and Lesbian: 0%  Bisexual: 0%  Don’t Know 4.4%  Prefer not to say: 4.4% |
| Age  >55  45-54  40-44  <40  not disclosed | 43.3%  4.3%  8.7%  43.5%  16.7% |
| Ethnicity  Black (Caribbean/African/British/Other)  Asian (British/Indian/Bangladeshi/Pakistani/Chinese/Other)  Mixed/Multiple (Caribbean/African/ Asian/ Other)  White (British/ Irish/Gypsy or Traveller/Other)  Other ethnic groups | 56.5%  21.7%  4.3%  8.7%  8.8% |
| Religion  Christian  Muslim  Hindu  Sikh  No religion  Others  Prefer not to answer | 39.1%  21.7%  4.4%  8.7%  13%  4.4%  8.7% |

|  | Pre-campaign uterine cancer awareness |
| --- | --- |
| **Do you know of any warning signs of womb cancer?**  Yes  No  **Free Text**  Bleeding  Pain  No Text | 43.5%  56.5%  61.5%  23.5%  15% |
| **If you had a symptom that you thought might be a sign of womb cancer, how soon after it started would you contact your doctor to make an appointment?**  1-2 weeks  Immediately  1-6 months  Had a condition  No comments | 17.4%  52.2%  13%  8.7% “Only if I could be guaranteed to see a doctor of the female sex”  8.7% |
| **In the following year, who do you think is most likely to develop womb cancer in the UK?**  A person aged 20 to 29 years  A person aged 30 to 49 years  A person aged 50 to 69 years  A person aged 70 or over  Womb cancer is unrelated to age | 4.4%  13%  26%  4.4%  52.2% |
| **What things (risk factors) do you think affect a person’s chance of developing womb cancer?**  Don’t Know  Know  **Most common**  Older age  Obesity  Smoking  Genetics  Contraceptive pills | 43.5%  56.5%  12.2%  12.2%  12.2%  7.3%  7.3% |
| **How confident are you that you would recognise womb cancer symptoms?**  Very confident  Not very confident  Fairly confident  Not at all confident | 8.7%  26.2%  17.3%  47.8% |
| **Do you think that cervical smear test screens for womb cancer?**  Yes  No  Don’t Know | 34.8%  56.5%  8.7% |
| **The following may or may not be warning signs of womb cancer. Whether you think each of the following is a sign of womb cancer.** | |
| **Vaginal bleeding between periods:**  Yes  No  Don’t Know | 69.6%  8.7%  21.7% |
| **Persistent lower back pain:**  Yes  No  Don’t Know | 47.8%  21.7%  30.5% |
| **Persistent abdominal pain:**  Yes  No  Don’t Know | 65.2%  8.7%  26.1% |
| **Persistent vaginal discharge that smells unpleasant:**  Yes  No  Don’t Know | 34.8%  21.7%  43.5% |
| **Discomfort or pain during sex:**  Yes  No  Don’t Know | 52.2%  21.7%  26.1% |
| **Menstrual periods that are heavier or longer than usual:**  Yes  No  Don’t Know | 43.5%  17.4%  39.1% |
| **Bowel Issues**  Yes  No  Don’t Know | 26.1%  17.4%  56.5% |
| **Vaginal bleeding after the menopause:**  Yes  No  Don’t Know | 65.2%  8.7%  26.1% |
| **Blood in stool or urine:**  Yes  No  Don’t Know | 43.5%  26.1%  30.4% |
| **A lump or swelling in your tummy or pelvis:**  Yes  No  Don’t Know | 60.9%  4.3%  34.8% |
| **Losing weight without trying:**  Yes  No  Don’t Know | 43.5%  13%  13.5% |
| **The following things (risk factors) may or may not increase a person’s chance of developing womb cancer. Some of these are correct, and some of these are myths. How Much do you agree that each of these can increase a person’s chance of developing womb cancer?** | |
| **Smoking cigarettes:**  Strongly agree  Agree  Not sure  Disagree  Strongly disagree | 30.4%  34.8%  26.1%  0%  8.7% |
| **Never using the contraceptive pill:**  Strongly agree  Agree  Not sure  Disagree  Strongly disagree | 0%  8.7%  34.8%  34.8%  21.7% |
| **Not having children:**  Strongly agree  Agree  Not sure  Disagree  Strongly disagree | 4.4%  17.4%  30.4%  21.7%  26.1% |
| **Being overweight**  Strongly agree  Agree  Not sure  Disagree  Strongly disagree | 30.5%  17.4%  43.5%  4.3%  4.3% |
| **Having Polycystic ovary syndrome (PCOS)**  Strongly agree  Agree  Not sure  Disagree  Strongly disagree | 8.6%  34.8%  47.8%  4.4%  4.4% |
| **Going through the menopause over the age of 55**  Strongly agree  Agree  Not sure  Disagree  Strongly disagree | 8.7%  17.4%  56.5%  13%  4.4% |
| **Having Diabetes:**  Strongly agree  Agree  Not sure  Disagree  Strongly disagree | 4.4%  17.4%  65.2%  8.7%  4.3% |
| **Having many sexual partners:**  Strongly agree  Agree  Not sure  Disagree  Strongly disagree | 4.4%  13%  43.5%  30.4%  8.7% |
| **Having breast cancer treatment (Such as Tamoxifen):**  Strongly agree  Agree  Not sure  Disagree  Strongly disagree | 13.1%  8.7%  69.6%  4.3%  4.3% |
| **Rarely exercising:**  Strongly agree  Agree  Not sure  Disagree  Strongly disagree | 8.7%  26.1%  43.4%  8.7%  13.1% |
| **Having relatives with bowel cancer or womb cancer:**  Strongly agree  Agree  Not sure  Disagree  Strongly disagree | 21.7%  56.5%  17.4%  0%  4.4% |
| **Hormone replacement therapy:**  Strongly agree  Agree  Not sure  Disagree  Strongly disagree | 8.7%  21.8%  60.9%  4.3%  4.3% |

**Supplementary data 5:**  **Detailed RE-AIM measures results**

| Dimension | Outcome |
| --- | --- |
| **Reach** | 1. **Event attendees**  - 88% were female and were over 45 years old. - 85.7% self-identified as being of Black or Asian ethnicity.  1. **Interviewees**  \| Common Themes \| Quotes \| \| --- \| --- \| \| Female Gender \| *"There's 28 ladies there. So this one I think was part of a menopause cafe. So this talk went really well. There was lots of engagement with women."* \| \| Ethnicity \| "*And I think people of different ethnicities were engaged, which was also really positive"*  *"There was 28 Black, Asian and White women. So it was a combined one."* \| \| Difficulty in penetrating the community \| *"I think there were a few challenges in getting people like local women involved in it and we had quite a small focus group,.."*  *"..most challenging was actually trying to get into these community places. So it took a lot of emails, a lot of once you did get through to them and you have to have a core to even pitch the talk because they don't know what you're asking them for. So it was a lot of time to even just get in and do this, but I don't think that you can change that. I think people are just going to be like that all the time."* \| |
| **Effectiveness** | 1. **Event attendees**  - 92.8% reported that they found the presentation easy to understand - 97.6% reported that information on the symptoms was adequate. - 92.8% felt encouraged to talk to a healthcare professional. - 94% were encouraged to speak to their friends and family.   **Free text**   \| - : “Very*helpful and learned a lot with this session”,* “*helped a lot”,*and another added, “*very well delivered”.* - *It makes me more aware of what is happening in my body”.* - *“Understanding symptoms can talk to daughter, one has heavy bleeding, happy to know all information, can talk to the carer, explain in detail about cancer.”* \| \| --- \|  1. **Interviewees (YNTK team)**  \| Common Themes \| Quotes \| \| --- \| --- \| \| Addressing misconception and Gynaecological cancers \| *"One point that was raised or that was picked up was how intrigued the women were about the cervical screening test not being a test for womb cancer. So very early on in our session, this is a very first session that we did and we realised at this point that women were under the impression that the smear test tested for womb cancer."*  *"very informative session and the takeaway for me was that cervical screening is not the same as screening for womb cancer. "* \| \| Insight of cultural influence \| *“There's a feeling that having bleeding is associated with fertility and being fertile is very important for you know how women feel about themselves, and therefore it's something to be welcomed rather than something to be wary of. If you have bleeding after the menopause."*  *"..but we've had things like you know, people weren't aware that things like so for example post-menopausal bleeding, they thought some ladies thought was normal, it was a normal thing to happen."* \| \| Transfer of knowledge within the community \| *"Sure. So I think a lot of women said what a useful time they had spent that they've really enjoyed it, that they had learnt a lot and that they would share the learning with their, you know, friends and family."*  *"they said they were going to share the message. We gave out leaflets so they said they were going to take it to maybe workplaces or give it to friends and family as well."*  "*So the highlights that were noted were that some of the leaflets were taken and put into a staff room by one of the ladies that she works in a school in [….]. A few women took a few women took extra leaflets to share with family and friends, especially Gujarati leaflets"* \| |
| **Adoption** | 1. **Interviewee (YNTK team)**  \| Common Themes \| Sub-themes \| Quotes \| \| --- \| --- \| --- \| \| Challenges \| Raising awareness and bringing the community together \| *“I think the campaign faced was being really local and having a local presence and reaching people in the community. I think that was done quite well, but we wished we'd done more. You know, we've sort of adverts, posters, things out and about in the community to drive people, to get the sessions, you know, to raise awareness.”*  *"The challenges were about how do we, who do we engage with. In terms of where do we put this information, how do we present this information and what information is it that's needed and how do we bring these people together? Well, where do we bring them together?”* \| \| Language as a barrier \| *"I've got the Flyers and the the assets in different languages, but where English is not their language, I felt maybe a translator would have been a good idea and look at because you want to have a really good impact. You want to have a full impact of the discussion. I think that language is one of the biggest barriers. And one of the biggest challenges,"*  *" I suppose, because it's the people we're focusing on, there's a lot of like women from South Asian backgrounds and that they don't always speak English as the first language, so unless we've got a translator that's always going to be an issue.”* \| \| Logistic challenges \| *“There'd been a Quran class before this, so there was13 Bengali ladies already gathered. And so we arranged for the 1 1/2 hour session, but they sort of abruptly told us that they needed to end after an hour because we always allow like 2 hours for a session to allow for set up and Q&A. But they had a fixed time. And so that was something that we hadn't really navigated before”* \| \| Communication \| Multiple media \| *“I had that prop in my set because people see the anatomy, like, you know how you've got, like, the womb. And then there's the cervix in there. So you sort of see that on a biology diagram when you're at school and, you know, if you Google the guy in anatomy.* *But I don't think you sort of see it as like that little neck of the womb”* \| \| Speaker’s delivery skills \| “*I had, you know, really good feedback that people had had enjoyed it. And I think the person delivering the that the talks was very gifted with. I think she's also a stand-up comedian and so had you know had a great a great way of communicating with her audience which I think was really positive.”*  *“I thought the delivery of the session, the talk was excellent. I mean, she really does touch on the subject really well and it's very informative. I think the informal sense of delivery is also a very nice way of informing these people, these women. I think.”* \| \| Shared experience \| *“one woman spoke about their own journey with cancer and it was very, very heart wrenching or what was beautiful is that all the different women on the call who didn't even know this woman very well. All came to support her and was like when you have your operation you can come and stay with me. I'll look after you. And if it felt like we've brought a community together to speak about an issue that they may not have spoken about with each other or have been expressed, especially this woman, she seems quite isolated or she didn't have a lot of family and friends and now”* \| \| Translation \| "*So if there was a limitation in the general groups level of English, then person she was liaising with, would, you know, speak to somebody from the group who did have a higher grasp of of English and they would then sort of translate between myself, either summarising every few slides or slide by slide, you know and I would go at a slower pace or whatever worked for the group. So yeah, that was a sort of something that came up sometimes and and not always."* \| \| Cultural differences \|  \| “*So that was one difference. And I think that's reflective of like the cultural differences as well, because the Somali ladies were just happy to talk amongst themselves and it was absolutely fine.”*  *“Whereas I feel and I understand that Indian women are very private about things, but also when it comes to like things that are taboo like this or sensitive, then the last thing they want to do is to stop telling everyone in the room because it is about how you. It's like your public appearance. You're going to see these people again and you don't really want them to know, oh, this is wrong with her. And you know everything else. So that that was the big difference. But I'm glad that we were able to build in extra time towards the end. So yeah, that's been the major difference.”* \| |
| **Implementation** | 1. **Event attendees**  - 89.7% felt that the location of the talk was convenient. - 94.1% enjoyed the talk. - 91.2% felt that the talk was appropriate and easy to understand. - 91.2% reported that the length of the session was appropriate.   **Free text**   \| - “very helpful and learn a lot with this session” - “very understandable, I will share with known and family” - “I learnt a lot about cancer” \| \| --- \|  1. **Interviewee (YNTK team)**  \| Common Themes \| Quotes \| \| --- \| --- \| \| Enjoyed \| *“I enjoyed the most was that people of all backgrounds all come or you know, everybody coming together way or from and who are you can have a laugh about the same sort of stuff. Like when I get my donut cushion out too.”*  *“I really like the fact that. People have come together”*  *“I just feel so proud and so privileged and so blessed to be able to do what I do. It's reflected in the feedback that I've received”* \| \| Positive feedback \| *"It was all positive. It was all. I always left feeling like the session had gone well and that we'd given information to people that didn't have the information before, which is obviously the whole point."*  *"Everyone was grateful for the presentation and their increased knowledge was reflected in the evaluation feedback. There wasn't really much Q&A besides the couple of ladies that were talking about the GP. But All in all, I was quite surprised at how well that session went. So that was that one. was the other online one that we ran again"* \| |
| **Maintenance** | 1. **Event attendees**   **Free text**   \| - *“didn’t know about the bleeding periods can mean that in between periods can mean that there is an issue, explained it really well, want more of these session please.”* \| \| --- \|  1. **Interviewee (YNTK team)**  \| Common Themes \| \| Quotes \| \| --- \| --- \| --- \| \| Wanting to continue \| *“We've spoken to quite a lot of other cancer alliances. Around the country, as well as just London, and they all thought the campaign was really good and, you know, really see it as something they'd like to aim towards or replicate. So we've had, you know, we've had conversations, I guess with other industry rather than the community side that I've had personally, and they've all been really positive”* \| \| \| Transferring the learning and experiences \| *“I mean, we're obviously thinking about phase two now and thinking about what else, what other cancers, get lady cancers, can we include, we're in discussion with that already.”*  *“As it is, I think it's it works really well. What we're thinking about for next year is actually what's come out of this is learning from what from what we've learnt and what we've been picking up is actually there's a lot a lack of awareness for other cancers, other bit like gynae cancers as well. So we're thinking of kind of how we can expand it. While keeping it as a, you need to know because keeping that tagline because I think that works, and but how we can expand it to talk about a bit more in depth about maybe ovarian cancer?”* \| \| |
